# Emerging multimodality imaging techniques for the pulmonary circulation

**DOI:** 10.1183/13993003.01128-2024

**Published:** 2024-10-31

**Authors:** Sudarshan Rajagopal, Harm J. Bogaard, Mohammed S.M. Elbaz, Benjamin H. Freed, Martine Remy-Jardin, Edwin J.R. van Beek, Deepa Gopalan, David G. Kiely

**Affiliations:** 1 Department of Medicine, Duke University School of Medicine, Durham, NC, USA; 2 Department of Pulmonology, Amsterdam University Medical Center, Location VU Medical Center, Amsterdam, The Netherlands; 3 Department of Radiology, Northwestern University Feinberg School of Medicine, Chicago, IL, USA; 4 Feinberg School of Medicine, Northwestern University, Chicago, IL, USA; 5 IMALLIANCE-Haut-de-France, Valenciennes, France; 6 Edinburgh Imaging, Queens Medical Research Institute, University of Edinburgh, Edinburgh, UK; 7 Department of Radiology, Imperial College Healthcare NHS Trust, London, UK; 8 Sheffield Pulmonary Vascular Disease Unit and NIHR Biomedical Research Centre Sheffield, Royal Hallamshire Hospital, Sheffield, UK

## Abstract

Pulmonary hypertension (PH) remains a challenging condition to diagnose, classify and treat. Current approaches to the assessment of PH include echocardiography, ventilation/perfusion scintigraphy, cross-sectional imaging using computed tomography and magnetic resonance imaging, and right heart catheterisation. However, these approaches only provide an indirect readout of the primary pathology of the disease: abnormal vascular remodelling in the pulmonary circulation. With the advent of newer imaging techniques, there is a shift toward increased utilisation of noninvasive high-resolution modalities that offer a more comprehensive cardiopulmonary assessment and improved visualisation of the different components of the pulmonary circulation. In this review, we explore advances in imaging of the pulmonary vasculature and their potential clinical translation. These include advances in diagnosis and assessing treatment response, as well as strategies that allow reduced radiation exposure and implementation of artificial intelligence technology. These emerging modalities hold the promise of developing a deeper understanding of pulmonary vascular disease and the impact of comorbidities. They also have the potential to improve patient outcomes by reducing time to diagnosis, refining classification, monitoring treatment response and improving our understanding of disease mechanisms.

## Shareable abstract

**Innovative imaging can enhance our understanding of pulmonary vascular disease mechanisms, refine classification, improve outcomes by reducing time to diagnosis, aid treatment decision-making and provide a holistic approach to assess impact to therapies.** https://bit.ly/3RvMWTQ

## Utility of imaging in the future

The current World Symposium of Pulmonary Hypertension (WSPH) clinical classification of pulmonary hypertension (PH) relies on a combination of haemodynamic and patient clinical characteristics for determining a patient's specific clinical group [1]. Although specific clinical and haemodynamic criteria have been developed to diagnose pulmonary arterial hypertension (PAH), they are limited in their utility in the setting of concomitant left heart disease (group 2 PH) or lung disease (group 3 PH). The presence of pre-capillary PH at right heart catheterisation (RHC) may be reactive in the setting of an elevated pulmonary capillary wedge pressure or may represent pulmonary vascular disease with obstructive vasculopathy of the pulmonary arterioles that results in right heart failure and death [2]. Some patients fit into multiple WSPH groups [3] and decisions regarding their diagnosis and treatment can be challenging. Our current clinical assessment relies on indirect or surrogate assessments of pulmonary vascular remodelling and their sequelae, including exercise capacity (from a 6-min walk distance) haemodynamics (from invasive RHC), right ventricular function (from echocardiography or cardiac magnetic resonance imaging (CMR)) and biomarkers of right heart strain such as N-terminal pro-brain natriuretic peptide (NT-proBNP). As the majority of patients who are now evaluated for suspected PH are older, with common cardiac and pulmonary comorbidities [4], these conventional clinical and haemodynamic criteria are frequently inadequate for diagnosing and monitoring patients [5]. The inability to visualise pulmonary vascular remodelling can make it difficult to diagnose and monitor specific forms of PH such as PAH, as many heart and lung conditions can impact these surrogate markers.

**FIGURE 1:**
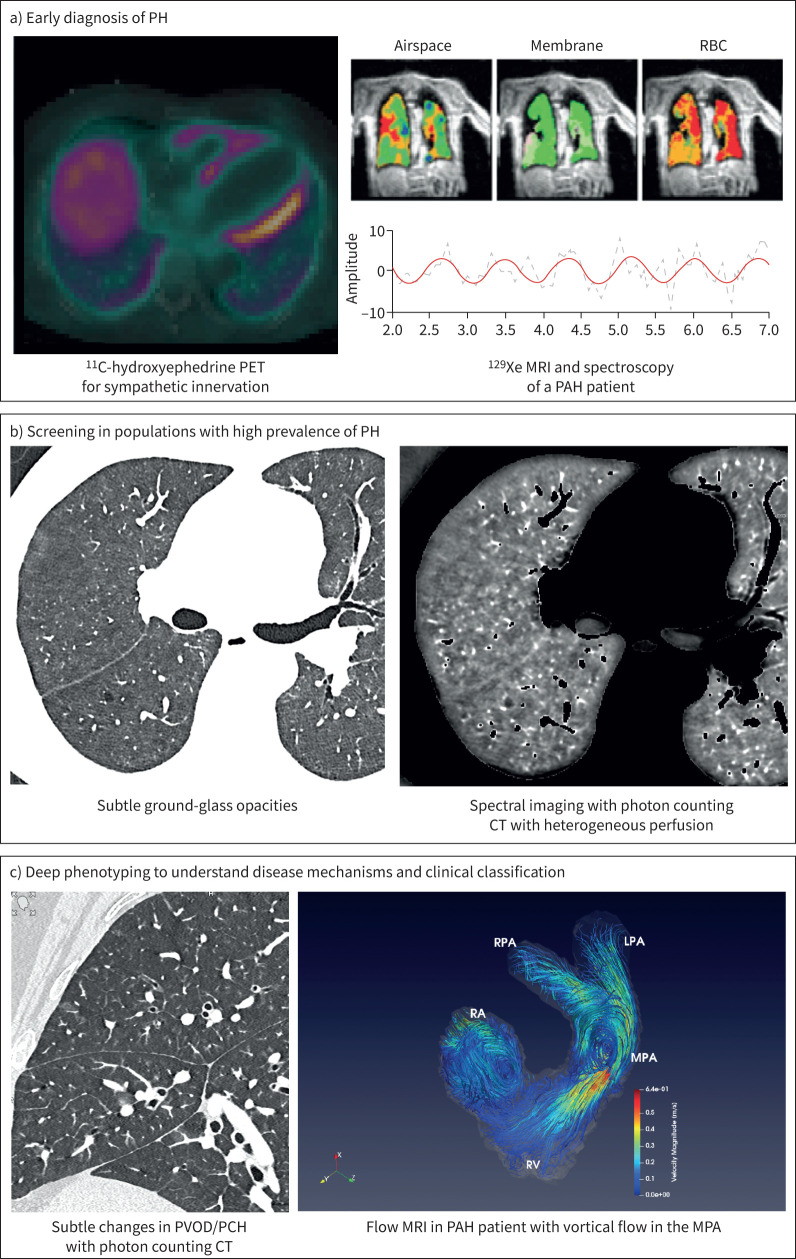
Advances in pulmonary vascular imaging in the diagnosis and classification of pulmonary hypertension (PH). a) Early diagnosis of PH in at-risk groups. b) Screening in populations with a high prevalence of PH. Chest computed tomography (CT) angiography in a 71-year-old male with systemic sclerosis and PH demonstrating (left panel) subtle nodular ground-glass opacities and (right panel) spectral imaging with photon-counting CT demonstrating heterogeneous perfusion. c) Deep phenotyping to understand disease mechanisms and clinical classification: (left panel) subtle changes in pulmonary veno-occlusive disease (PVOD)/pulmonary capillary haemangiomatosis (PCH) with photon counting CT; (right panel) three-dimensional (3D) blood flow visualisation (Streamlines) in the right heart from 4D flow magnetic resonance imaging (MRI) in a 39-year-old female with pulmonary arterial hypertension (PAH), showing vortical (swirling/rotating) flow in the main pulmonary artery (MPA) and right ventricle (RV) during late systole. Colours indicate flow velocity magnitude (range is 0–0.64 m·s^−1^). ^11^C: carbon-11; PET: positron emission tomography; ^129^Xe: xenon-129; RPA: right pulmonary artery; LPA: left pulmonary artery; RA: right atrium.

**FIGURE 2:**
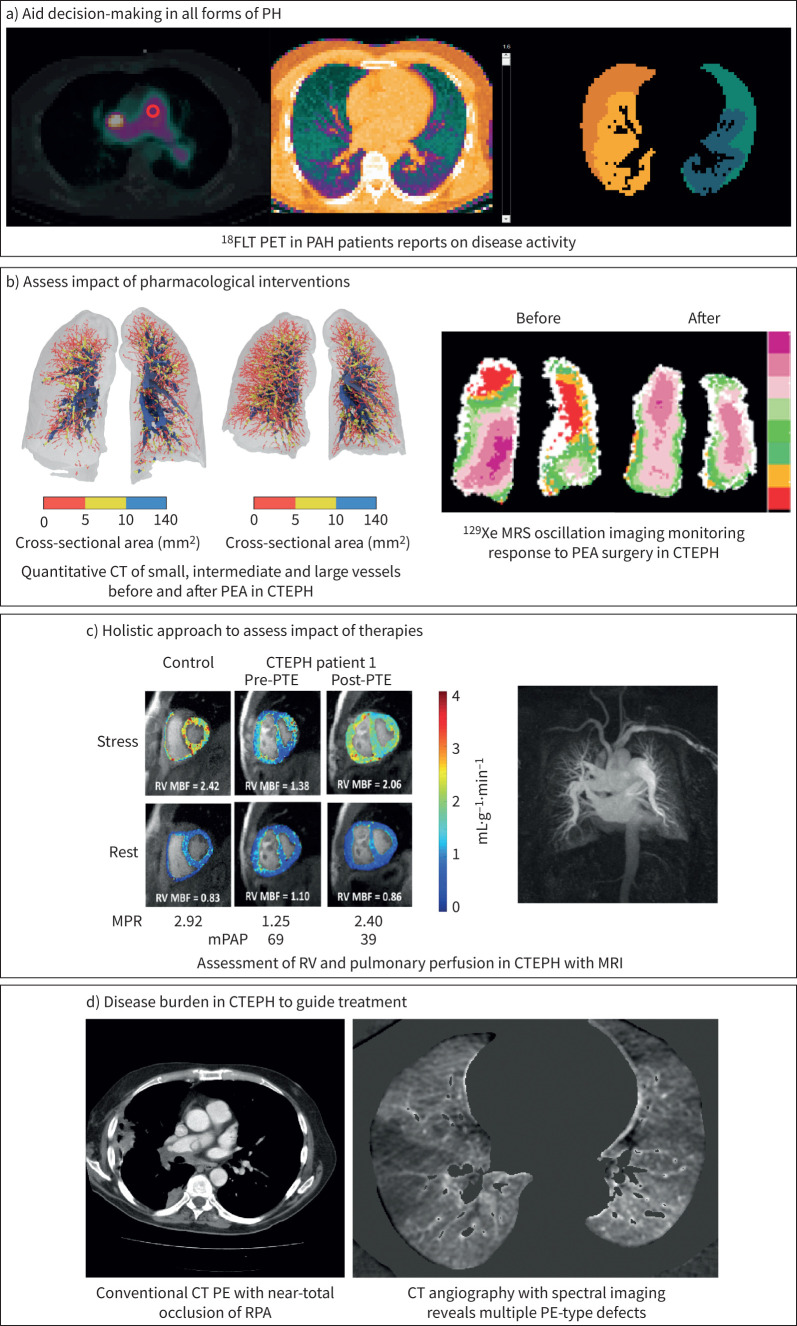
Advances in pulmonary vascular imaging in guiding assessing treatment of pulmonary hypertension (PH). a) Aid decision-making in all forms of PH. 3′-deoxy-3′-(^18^F)-fluorothymidine (^18^FLT) positron emission tomography (PET) scanning is increased in some patients with pulmonary arterial hypertension (PAH), which could signal a better treatment response to antiproliferative drugs. b) Assess impact of pharmacological interventions. (Left panel) Three-dimensional reconstruction of the macroscopic pulmonary vessels detected using a fully automated quantitative computed tomography (CT) pipeline software (Fluidda, Inc., USA). Small vessels (<5 mm^2^ in cross-sectional area) are depicted in red, intermediate vessels (>5 mm^2^ and <10 mm^2^) are depicted in yellow, and large vessels (>10 mm^2^) are depicted in blue. On the baseline CT (left) of a patient with chronic thromboembolic pulmonary hypertension (CTEPH) there is dilatation of large proximal vessels and loss of smaller vessels. Following successful pulmonary endarterectomy (PEA) (right), there is reduction in the larger vessels and significant increase in the distribution of the smaller vessels. (Analysis and image courtesy of Ben R. Lavon and Fluidda, Inc.) (Right panel) Xenon-129 (^129^Xe) magnetic resonance sounding (MRS) oscillation imaging identifies regions with decreased perfusion (red) at baseline in a patient with operable CTEPH (before), with significant improvement after PEA surgery (after). c) Holistic approaches to assess impact of therapies. (Left panel) Magnetic resonance imaging (MRI) assessment of changes in right ventricular (RV) perfusion in CTEPH with PEA and (right panel) magnetic resonance angiography of disease burden in CTEPH. d) Disease burden to guide treatment. (Left panel) conventional CT pulmonary embolism (PE) demonstrating near-total occlusion of the right pulmonary artery. (Right panel) CT angiography with spectral imaging providing iodine maps that reveal multiple PE-type defects. RV: right ventricle; MBF: myocardial blood flow; MPR: multiplanar reformation; mPAP: mean pulmonary artery pressure; RPA: right pulmonary artery.

**FIGURE 3:**
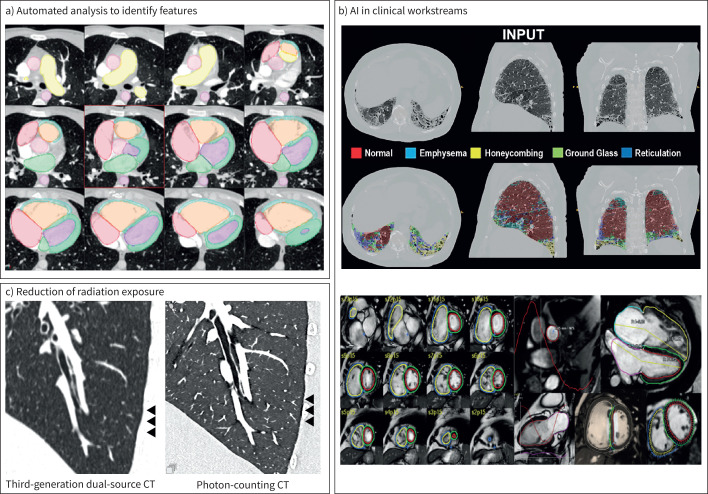
Technical advances and application of artificial intelligence (AI) in imaging in patients with pulmonary vascular disease. a) AI extraction of pulmonary hypertension (PH) features from standard computed tomography pulmonary angiography images including pulmonary artery dimensions, right ventricular hypertrophy and cardiac volumes to allow for fully automated PH diagnosis with potential to aid earlier diagnosis of PH. Reproduced and modified from [6] with permission. b) AI implementation in clinical workstreams. (Upper panel) Segmentation of lung parenchyma in patient with PH to aid classification; normal parenchyma (red), emphysema (teal), honeycombing (yellow), ground glass (green) and ground glass with additional reticulation (blue) allowing for improved phenotyping of patients with PH (group 1 *versus* group 3) (performed using the approach in Dwivedi *et al.* [7]). (Lower panel) Example of AI automated analysis incorporated into clinical workstreams with measurements of cine and flow sequences from cardiac magnetic resonance imaging allowing for rapid and fully automated quantification of metrics in patients with PH. Adapted from Alabed *et al.* [8]. c) Reduction to radiation exposure with computed tomography (CT). Compared to a conventional energy-integrating detector CT (third-generation dual-source CT) (left panel), the photon-counting CT (right panel) reduces radiation exposure with superior visualisation of small vessels such as supernumerary arteries (arrowheads).

Ideally, newer imaging modalities could aid diagnosis, classification and treatment of patients with PH (table 1). Such techniques would identify early disease or pre-clinical disease in patients who are at risk of PAH (such as patients with systemic sclerosis or *BMPR2* mutation carriers). These approaches could also be used for deep phenotyping of patients and aid classification (figure 1) [5], and ideally, in the future, obviate the need for invasive RHC for diagnosis. Imaging could also aid in treatment decision-making in patients with PH (figure 2), providing more sensitive biomarkers for monitoring response to therapy. In addition, these new imaging approaches would provide technical improvements to current standard of care and inform the development of new algorithms to synthesise clinical and imaging information to guide treatment (figure 3). Together, these current unmet needs emphasise the necessity for novel and robust imaging for diagnosis, classification and treatment guidance given the complexity of PH and the heterogeneity of pulmonary vascular involvement.

**TABLE 1:** Important challenges for future imaging in pulmonary hypertension (PH)

**Diagnosis and classification**	Early diagnosis of PH in patients presenting with unexplained breathlessness
	Screening those with a high prevalence of PAH (*e.g.* systemic sclerosis and *BMPR2* mutation carriers)
	Deep phenotyping to improve our understanding of disease mechanisms and the clinical classification of PH
	Ultimately replace invasive techniques such as RHC
**Treatment**	Aid treatment decision-making in patients with all forms of PH
	Use imaging to assess the impact of pharmacological interventions on remodelling of the distal pulmonary vasculature by combining and developing structural techniques (CT) with functional imaging (MRI)
	Use imaging to provide a more holistic approach to assess the impact of therapies (gas exchange, pulmonary vasculature and cardiac)
	Assessment of disease burden in patients with CTEPH to guide decisions regarding surgery, balloon pulmonary angioplasty or medical therapy
**Technical**	Automated analysis of routinely performed imaging (*e.g.* chest radiography, echocardiography, CT imaging, MRI) to identify established imaging features of PH and using AI approaches to identify novel imaging features
	Incorporate AI into clinical workstreams to aid decision-making by combining automated analysis of routinely performed imaging tests and integrating those results with clinical features and the results of commonly performed studies
	Reduce radiation exposure by developing new CT techniques and using nonionising radiation approaches

PAH: pulmonary arterial hypertension; RHC: right heart catheterisation; CT: computed tomography; MRI: magnetic resonance imaging; CTEPH: chronic thromboembolic pulmonary hypertension; AI: artificial intelligence.

## Computed tomography

Technological advances in computed tomography (CT) are expected to play an important role in the evaluation of PH, ranging from wider clinical implementation of already available tools to developments in morphofunctional approaches.

### Currently available clinical imaging tools

An automated extraction of the smallest peripheral blood vessels is accessible from noncontrast and contrast-enhanced chest CT examinations. This approach can provide information on the pulmonary vessel radiographic appearance described as vascular pruning. Synn *et al.* [9] reported that more severe pulmonary vascular pruning on CT was associated with greater small-vessel pulmonary arterial remodelling on histology. The ratio of the number of arteries over veins with diameters between 6 and 10 mm was also found to be significantly correlated with prognostic markers in PH and predicts low and high mortality risk [10]. Therefore, CT-derived parameters may be useful as additional criteria for the noninvasive risk stratification in pre-capillary PH. More sophisticated analysis of blood vessel morphology, such as vessel tortuosity *(i.e.* a measure of twistedness of blood vessels) and three-dimensional (3D) fractal dimension (*i.e.* a parameter measuring the complexity of the lung vascular tree) of segmented lung vessels have already been proposed as noninvasive tools to evaluate the severity of pulmonary hypertension [11].

The availability of spectral imaging, relying on the possibility of simultaneous acquisition of several CT datasets at different mean X-ray energies, represents a major development for the evaluation of PH (figure 1b). Until recently, only dual-energy CT was used using different technologies such as dual-source, dual-layer and rapid tube voltage switching. CT systems utilising spectrally sensitive photon-counting detectors are now available (figure 1c and figure 3c). Among the various image sets that can be generated with spectral imaging, the possibility of creating iodine maps, currently accepted as surrogate markers of lung perfusion, is of particular interest in the diagnostic approach of PH aetiology. Although commercially available for more than two decades, CT lung perfusion has remained in the position of an emerging imaging modality owing to the lack of consensus on the scanning protocols to best capture changes at the level of lung microcirculation and the relatively limited availability of corresponding CT equipment and analysis software.

In the context of chronic thromboembolic pulmonary hypertension (CTEPH), good agreement has been reported between CT lung perfusion and scintigraphy for the detection of perfusion defects (figure 2d) [12]. Dual-energy CT-depicted perfusion defects have also shown good correlation with haemodynamic estimates of PH severity [13, 14]. Compared to ventilation/perfusion (*V*′/*Q*′) scintigraphy, high diagnostic values of CT perfusion have been reported, with sensitivities and specificities ranging from 97% to 100% and from 86% to 92%, respectively [15–17]. In a recent meta-analysis of single-centre studies gathering 734 patients with suspected CTEPH, the diagnostic performance of CT pulmonary angiography (CTPA) and lung perfusion was analysed separately [18]. The pooled sensitivity, specificity, positive predictive value (PPV), negative predictive value (NPV), accuracy and diagnostic odds ratio (DOR) estimates for CTPA in the detection of CTEPH were 0.98, 0.99, 0.94, 1.00, 0.96, 0.9 and 292, respectively. Evaluation of perfusion changes yielded pooled estimates for sensitivity, specificity, PPV, NPV, accuracy and DOR of 0.99, 0.84, 0.79, 0.98, 0.89, 0.89 and 98, respectively, across four studies with 278 patients.

When the analysis of iodine maps is combined with standard CT images of pulmonary arterial morphology, dual-energy CT reaches a sensitivity and specificity of 100% for a diagnosis of CTEPH [15, 16, 19]. These promising results require validation on a larger scale, but reinforce the growing importance of modern CT techniques in the diagnosis of CTEPH [19, 20]. Moreover, the patterns of lung perfusion demonstrated with dual-energy CT can differentiate PAH from peripheral forms of CTEPH, concordant with *V*′/*Q*′ scintigraphy [21]. A variable association of perfusion abnormalities have been reported in pulmonary veno-occlusive disease (PVOD)/pulmonary capillary haemangiomatosis (PCH) with lobular and PE-type perfusion defects larger than a sub-segment depictable in both PAH and PVOD/PCH patients [22]. It is also promising to consider that CT lung perfusion could help detect lung microvasculopathy in the absence of morphological changes and prior to the development of PH, as recently reported in the context of systemic sclerosis (figure 1b) [23]. There is a need for further development of regional quantitative approaches to assess pre- and post-therapeutic changes, highlighted in the context of CTEPH, but also relevant in other forms of PH therapeutic approaches [24–26].

### CT morphometry

CT-based computational vascular morphometry, a technique to profile the intraparenchymal blood volume and geometry, can be useful for noninvasive assessment of vascular remodelling. Using artificial intelligence (AI) algorithms, it is possible to extract the volume of blood distribution in the pulmonary vessels of varying sizes from routine CT scans. The blood volume is computed as a function of the cross-sectional area of the vessel [27]. The ratio of the small vessel volume to total blood volume has been proposed as a CT measure of vascular remodelling in PH. The loss of distal vasculature quantified as “pruning” on CT correlates histologically with vascular density loss [28], increased small vessel wall thickening and reduction in luminal cross-sectional area [9]. CT-based pruning quantification has been linked to worse clinical outcomes in a variety of pulmonary vascular disorders such as smoking, asthma, COPD, PAH and CTEPH (figure 2b) [29–37].

In PH, different patterns of morphometric abnormalities have been reported. Increased arterial, but not venous tortuosity has been shown to be a feature of PAH as well as CTEPH [36, 38]. In a small cohort of 42 PAH patients and 12 patients with exercise-induced PH, Rahaghi *et al.* [38] showed that while distal arterial and venous pruning was present in both groups, proximal arterial dilatation was limited to the PAH cohort. This technique represents a unique quantitative method that may be useful in monitoring response to therapy, as was shown in a very recent study [39]; serial imaging in PAH patients treated with seralutinib, a novel inhaled kinase inhibitor with the potential to reverse remodelling, showed significant redistribution of pulmonary arterial blood vessel volumes to smaller vessels.

Fractal dimension (FD) analysis is another CT imaging-based technique that can be used to quantify morphological changes in the pulmonary vasculature. Fractal dimension of 2D projections of the pulmonary vessels in children with PH has shown negative correlation between lower FD and pulmonary vascular resistance (PVR) with poorer survival [40]. However, Helmberger *et al.* [11] used FD analysis using 3D projections in adults with PH and failed to show a significant correlation between FD and haemodynamic parameters, whereas the distance metric, a measure of the vascular tortuosity correlated with mean pulmonary artery pressure (mPAP) and PVR as well as World Health Organization functional class. Future larger scale validation is needed to determine whether CT-based morphometrics can play a definitive role in screening for pulmonary vasculopathy.

### Emerging approaches

The recent introduction of photon-counting-detector-CT technology (figure 3c) is expected to influence the noninvasive approach of pulmonary microvasculopathy, currently considered out of the scope of imaging. Evaluation of lung parenchymal structures with ultra-high resolution (UHR) makes subtle changes at the level of the secondary pulmonary lobules and lung microcirculation visually accessible, opening new options for synergistic collaborations between highly detailed morphology and AI. In the context of CTEPH, the well-described pathophysiological differences in lung microcirculation between areas of occluded and non-occluded pulmonary arteries could be approached on UHR lung images. This information could be combined to a more precise morphological analysis of peripheral pulmonary arteries in order to provide better delineation of target vessels before balloon pulmonary angioplasty (BPA).

The combination of multienergy perfusion imaging of the lungs with lobar segmentation can automatically visualise perfusion deficits, providing pixelwise quantification of the spatial extent of hypoperfusion [41]. The spatial extent of hypoperfusion and global hypoperfusion volumes was able to separate patients with CTEPH from controls and correlated with invasive PVR and change in PVR with surgery, even in patients with surgically defined segmental disease.

### Role of CT in phenotyping PH patients

PH is highly heterogenous. Hoeper *et al.* [42] highlighted the importance of phenotyping patients with idiopathic PAH (IPAH), with an IPAH lung phenotype characterised by a low diffusing capacity of the lung for carbon monoxide (*D*_LCO_) (<45%) and a smoking history having a similar demographic profile and survival to patients with group 3 PH. A significant proportion of these patients had minor lung disease on CT a feature that has previously been shown to negatively impact on survival in patients who meet the current diagnostic criteria for IPAH [43]. Therefore, being able to identify even minor lung disease in severe pre-capillary PH is important. Recent reviews have highlighted the different aspects of PH associated with COPD and interstitial lung disease (ILD) and patients with combined pulmonary fibrosis and emphysema (CPFE) [44, 45], and the potential for AI to improve phenotyping [46]. COPD and ILD have aspects in common, such as chronic hypoxaemia, vascular pruning and endothelial dysfunction; there are many other factors that are different, such as environmental and genetic influences as well as inflammation pathways, suggesting that the disorders should be studied as separate entities to improve outcomes [45]. Accurate classification of patients with lung disease remains challenging and the complexity of the CPFE subgroup has been highlighted elegantly by Cottin *et al.* [47]. AI has aided lung parenchymal assessment allowing accurate quantification of the degree of emphysema, fibrosis, ground-glass change and reticulation and has recently been shown to aid prognostic prediction in patients with group 3 PH [7] and may allow for improved stratification for entry into clinical trials. This interest in improved evaluation of PH complicating severe forms of COPD/ILD relies on the availability of new therapeutic options [47, 48] and it is hoped that improved phenotyping using CT will aid identification of patients who may be more likely to benefit from specific interventions.

## Magnetic resonance imaging

Magnetic resonance imaging (MRI) uses the magnetic spin of atoms to generate a signal, by placing the area of interest in a high magnetic field. Proton MRI applies to all techniques which use the proton atom as the basis of images, with or without the addition of signal enhancing contrast agents. Non-proton MRI applies to all techniques that use other atoms. This usually means extraneous agents, such as oxygen (which is paramagnetic), or more frequently helium-3 (^3^He), xenon-129 (^129^Xe) or phosphorus-31, which can all be introduced once energised using high-energy laser beams. The most commonly used methods require fast-spin echo sequences (turbo-spin echo, *etc*.), which allow visualisation of anatomical structures, including muscle, bone and soft tissues. In PH, sequences that utilise a gated spin-echo double inversion recovery (black-blood MRI) can be useful in identifying slow-flowing blood in the pulmonary vasculature. In a study of 233 patients with suspected PH, this sequence had an 86% sensitivity and 85% specificity for diagnosing PH [49]. Other sequences apply to increased emphasis on faster image acquisition and tend to be water based, such as half-Fourier acquisition single-shot turbo spin-echo. The lungs have a sparse proton environment combined with magnetic disturbances due to the many interfaces between air and the soft tissues of the lung, which makes direct visualisation of the lung challenging.

The use of intravascular contrast agents, most commonly gadolinium, can be used to assess the pulmonary vasculature. Magnetic resonance angiography provides high spatial resolution images of the pulmonary vasculature (figure 2c). It can help differentiate between different types of PH by identifying vessel pruning in PAH or signs of CTEPH, such as organised thrombotic material, vessel wall thickening and post-stenotic dilation [50–53]. By combining this technique with more speedy acquisition, it is possible to follow a contrast agent bolus from the venous phase, through the right heart into the pulmonary arteries, and then through the capillary phase in the pulmonary veins. This method, dynamic contrast-enhanced (DCE) MRI, allows direct visualisation of lung perfusion and can identify defects associated with various causes of PH [54]. DCE additionally provides quantitative analysis of lung perfusion by providing pulmonary blood flow, pulmonary blood volume and mean pulmonary transit time [55, 56]. Mean pulmonary transit time has diagnostic and prognostic importance in PAH and CTEPH as well as other lung diseases [57–59]. DCE is particularly adept at identifying disease progression as well as treatment response [60, 61]. In a recent study, DCE and dual-energy CT were comparable in the detection of lung perfusion improvement in patients with CTEPH post-pulmonary endarterectomy [26]. The use of other contrast agents that report on lung perfusion include ferumoxytol [62] and oxygen-enhanced MRI [63].

A more recent development has been a method which uses signal processing to subtract images during inspiration and expiration. This method, phase-resolved functional lung (PREFUL), is able to demonstrate magnetic resonance perfusion and magnetic resonance ventilation images without the need for extraneous contrast. PREFUL has been studied in a variety of pulmonary pathologies including PH and, overall, is a highly reproducible technique. However, it is susceptible to different field strengths and sequences, which can greatly impact the degree of perfusion abnormalities [64]. In a multicentre study evaluating this method in patients with suspected CTEPH, PREFUL was able to detect similar lung perfusion defects at multiple sites, but signal- and contrast-to-noise ratios were significantly different between study centres [65].

One of the challenges for new diagnostic approaches is the lack of robust evidence comparing diagnostic strategies and such studies are crucial to aid the development of evidence-based guideline recommendations [66]. CHANGE-MRI is a large prospective multicentre, comparative, phase III diagnostic European study examining whether functional MRI can serve as an equal rights alternative to *V*′/*Q*′-single-photon emission CT in a diagnostic strategy in patients with suspected CTEPH. It aims to enrol 1080 patients and will inform our approaches to the diagnostic assessment of CTEPH [67].

### 4D flow MRI

4D flow MRI permits direct *in vivo* visualisation and quantification of 3D velocities in the pulmonary artery and right heart chambers (figure 1c) [68]. It enables noninvasive derivation of advanced haemodynamic parameters, such as wall shear stress (WSS) [69], vortex flow [70, 71], vorticity [72, 73], kinetic energy [72], viscous energy loss [72] and pressure gradients [74]. In PH patients, 4D flow MRI demonstrated a linear correlation mPAP and vortex flow duration in the main pulmonary artery (MPA). A relative vortex flow duration in the MPA >14.3% of the cardiac cycle was diagnostic of PH with 97% sensitivity and 96% specificity [73]. While promising, vortex duration-based pressure estimation has several limitations including being capped at 79 mmHg [73], associated with MPA diameter [9, 12, 13], vortex flow was also reported in healthy volunteers [75], and the derived diagnostic threshold may be affected by scan parameters, mPAP cut-off, and the inability to differentiate between PH groups [76]. Hence, more work is needed to establish robustness and independent value of vortex duration over existing metrics. Vorticity in the main and right pulmonary arteries correlates with PVR in PH [77], while helicity in MPA, right pulmonary artery, and right ventricular outflow tract correlated with MPA stiffness and right ventricular (RV) function indices [73]. Likewise, retrograde flow onset in the MPA correlated with mPAP [78]. 4D flow MRI-derived WSS was lower in proximal pulmonary arteries in PAH patients compared to healthy controls [69] and correlated with markers of stiffness, including capacitance, distensibility and elasticity [69]. However, WSS and vorticity accuracy and reproducibility can be sensitive to 4D flow MRI scan resolution and noise [68].

While 4D flow MRI holds a strong promise for comprehensive noninvasive multiparametric haemodynamics evaluation throughout the right heart, its clinical translation is hindered by current limitations. Existing 4D flow MRI studies are often small and single-site, necessitating larger, multicentre investigations for robust PH diagnosis and group differentiation. 4D flow MRI complex analysis and underutilised data dimensionality call for innovative tools to enable robust full right-heart haemodynamic analysis. A recently proposed 4D virtual catheter technique integrating mathematical modelling and 4D flow MRI might hold promise for comprehensive haemodynamics evaluation in PH [72].

### Hyperpolarised gas

The noble gases ^3^He and ^129^Xe are isotopes which are capable of producing signal within an MRI environment once energised using laser optical pumping. This technology causes a net spin shift (hyperpolarisation) with a larger number of atoms becoming aligned within a magnetic field. Upon the introduction of radiofrequency pulses within the MRI scanner, the atoms will become randomly distributed again, and this process provides a signal which can be transformed into images. ^3^He has the ability to measure a number of key lung characteristics, including ventilation distribution, airspace size (at alveolar level) and local blood oxygen uptake within the lung. These technologies are all of potential relevance in the context of PH, depending on the type of PH (*e.g.* COPD and pulmonary vascular disease demonstrate markedly different findings).

Although early clinical applications focused on hyperpolarised ^3^He, new technologies combined with lack of access to ^3^He has prompted renewed interest in the use of ^129^Xe MRI. The diffusivity and frequency shifts of ^129^Xe allow a direct, quantitative assessment of airspace (ventilation distribution), transmembrane diffusivity (allowing an assessment of lung parenchyma) and red blood cell oxygenation through direct sampling of the ^129^Xe atom in different phases [79]. This allows the generation of quantitative maps to depict the distribution of ^129^Xe in airspaces, its uptake in lung parenchyma and its binding to haemoglobin in red blood cells [80, 81]. These physiological properties shed insight into the full lung function, from inhaled oxygen to oxygen transport, and also correlates with different types of flow disturbance in various causes of PH. Thus, ^129^Xe MRI/magnetic resonance sounding (MRS) metrics have the potential to differentiate between patients with different cardiopulmonary diseases.

Initial studies of ^129^Xe MRI have been promising, demonstrating different characteristics compared to other common heart and lung diseases. PAH has been associated with a unique ^129^Xe MRI signature compared to COPD, idiopathic pulmonary fibrosis and left heart failure (figure 1a) [82]. In early studies, oscillations in red blood cells amplitude and frequency appear to provide insight into pulmonary vascular *versus* interstitial origins of gas exchange impairment [83]. These differences in ^129^Xe MRI signatures are conceptually linked to changes in remodelling and fibrosis with these diseases. For example, the decreased ^129^Xe MRS oscillations (figure 2b) in PAH correlate with an increased pre-capillary resistance to blood flow (pre-capillary PH), compared to the high oscillations observed in left heart failure due to post-capillary PH. In a small study with same-day RHC, ^129^Xe MRI/MRS displayed good diagnostic accuracy in identifying patients with PAH [84]. These studies suggest that ^129^Xe MRI/MRS has the potential to address the current lack of disease-specific, noninvasive biomarkers for diagnosis and monitoring of PAH, but these metrics have yet to be rigorously evaluated in larger studies.

## Positron emission tomography/CT

### Fluorine-18 fluorodeoxyglucose

Fluorine-18 fluorodeoxyglucose (^18^F-FDG) is a radiolabelled glucose analogue and is the most common positron emission tomography (PET) radiopharmaceutical for qualitative and quantitative assessment of glucose metabolism. Coupled with a CT scanner, modern hybrid PET-CT systems offer structural and metabolic information that can be effectively used to identify sites of inflammation, infection and malignancy.

#### PAH

Small studies using ^18^F-FDG-PET imaging to evaluate pulmonary vascular remodelling and inflammation in PAH have shown mixed results. Aerobic glycolysis, characterised by the high rate of glucose consumption even in the presence of adequate oxygen (“Warburg effect”) is an inherent feature of cancer biology [85]; a similar glycolytic shift has also been observed in human PAH patients [86, 87]. Zhao *et al.* [88] performed dynamic ^18^F-FDG PET imaging with kinetic modelling in PAH patients (n=20) and showed significantly higher FDG uptake in the lungs of IPAH cohort when compared to healthy controls as well as higher rate of glucose phosphorylation (k3) in a proportion of IPAH patients. But the distribution of FDG within the lungs of IPAH cohort was nonuniform, a reflection of the heterogeneity of vascular abnormalities in this disease.

Cardiac alterations in glucose metabolism have been studied extensively and have shown an inverse correlation between RV ^18^F-FDG uptake and function in PAH patients [89–93]. Conversely, the pulmonary ^18^F-FDG uptake is less well investigated and available data do not show a firm relationship between lung FDG uptake and various clinical, laboratory and haemodynamic markers of PH severity and survival [94, 95]. In monocrotaline rat models, pulmonary ^18^F-FDG uptake has been shown to be reduced following dichloroacetate and tyrosine kinase inhibitors [88, 96]. This effect has also been corroborated by *in vitro* modelling using pulmonary fibroblasts isolated from IPAH patients; the hyperproliferative phenotype with upregulated glycolytic gene expression demonstrated reduction in ^18^F-FDG uptake by dichloroacetate and imatinib [88]. In a phase 1 clinical trial using dichloroacetate in IPAH, only patients who showed haemodynamic improvement had reduction in pulmonary FDG uptake whilst those without response had an increase in lung ^18^F-FDG uptake [97]. More recently, in a small study by Wang *et al.* [98], patients with systemic lupus erythematosus (SLE)-related PAH (n=14), ^18^F-FDG lung uptake showed correlation with SLE disease activity and immune/inflammatory status (C3 and C4) but not with pulmonary vascular haemodynamics or 6-min walk test.

While ^18^F-FDG-PET imaging holds promise for the assessment of pulmonary vascular remodelling and treatment evaluation, the clinical utility of increased pulmonary FDG-uptake as a solitary parameter is currently limited.

#### PH in association with lung disease

^18^F-FDG uptake is a marker of the global inflammatory response in the lungs and has been shown to be increased in in the context of pulmonary vascular remodelling in COPD [99] and ILD [100]. In a study involving 109 patients with end-stage lung disease (predominantly COPD and ILD) undergoing evaluation for lung transplant [101], increased ^18^F-FDG uptake was noted in the RV and lung parenchyma correlated with the presence and severity of PH. Moreover, the authors also report increased ^18^F-FDG uptake in central pulmonary arteries, which strongly correlated with mPAP. This differs from a previous study in IPAH patients [87] where there was no uptake in the large pulmonary arteries. Although it is tempting to attribute the difference in the pulmonary arterial uptake to the differences in the underlying pathophysiological mechanisms (WSPH group 3 *versus* 1), the results must be interpreted with caution, as these are single-centre studies with small patient cohorts with varying stages of disease.

Sarcoidosis is a multisystem disorder with the lung most commonly involved. ^18^F-FDG PET/CT plays a multifactorial role in the evaluation of sarcoidosis; it can define disease extent and inflammatory activity, provide biopsy guidance, aid prognostic evaluation and monitor treatment response [102, 103]. Sarcoid-associated PH may be due to multiple mechanisms including pre-capillary (reflecting pulmonary vascular involvement) or post-capillary. Patients with sarcoidosis and pulmonary fibrosis have the highest prevalence of PH [104]. However, there are no reliable tools to predict progression of sarcoid-associated pulmonary fibrosis. While fibrosis is not always metabolically inert, ^18^F-FDG-PET has shown promise as a marker for active pulmonary inflammation [105–107]. Adams *et al.* [108] demonstrated the diagnostic superiority of using volumetric PET/CT (*i.e.* the percentage of lung volume with increased metabolic activity or the average metabolic activity in the lung) in 35 patients with pulmonary sarcoidosis; quantitative analysis showed better correlation with conventional biomarkers for disease activity when compared with visual dichotomous interpretation as PET “positive or negative” or the use of traditional standardised uptake value (SUV_max_), as the latter only represents the maximum uptake within one voxel rather than global inflammation in the lung.

#### PH associated with pulmonary artery obstruction

^18^F-FDG PET/CT has the potential to differentiate between chronic thromboembolism and pulmonary artery intraluminal tumours and can help in staging of the malignancy [109–112]. Pulmonary arterial tumour is characterised by high cellularity and neovascularisation and hence generally shows high ^18^F-FDG uptake. The temporal evolution of the pulmonary arterial thrombus from acute to chronic phase involves dynamic changes in the cellular and inflammatory component and this is reflected in the variability in its ^18^F-FDG-uptake. A pooled analysis showed that a cut-off value of 3.3, SUV_max_ is accurate for distinguishing between pulmonary artery malignancy from thrombus [113]. However, there are case reports showing false negative ^18^F-FDG-PET in pulmonary artery tumours [114–117], attributable to various reasons such as low tumour-cell density, high mucous component, marked interstitial myxoid tissue, tumour necrosis, haemorrhage or calcification. Moreover, ^18^F-FDG PET/CT cannot discriminate between the intimal and intramural sarcoma subtypes. In addition, there are sparse data on the role of PET/CT for monitoring response to treatment. Future prospective studies are needed to validate the role of ^18^F-FDG PET/CT in pulmonary arterial malignancies.

Pulmonary artery vasculitis is usually present in the context of an underlying large-vessel systemic vasculitis such as Takayasu arteritis, giant cell arteritis, and Behçet's or its forme fruste Hughes–Stovin syndrome. Although rare, there are case reports of isolated pulmonary arterial vasculitis [118–121]. Early diagnosis of vascular inflammation is pivotal to reduce the risk of vascular complications. ^18^F-FDG PET/CT may be of synergistic value for optimal diagnosis, monitoring of disease activity, and evaluating damage progression in large vessel vasculitis [122], but it is important to remember that the accuracy of ^18^F-FDG PET/CT uptake in vasculitis declines after >3 days of high-dose steroid treatment [123, 124]. As inflammatory changes in the arterial wall usually precede structural abnormalities, FDG PET can show active pulmonary vascular inflammation at an earlier disease stage with the added benefit of whole-body assessment, and hence can be used to guide the need for treatment [125]. ^18^F-FDG PET/CT is also useful in the assessment of the clinical course of the disease and to detect relapsing/refractory disease [126].

IgG4-related disease (IgG4-RD) is an immune-mediated systemic fibroinflammatory disorder that can be associated with protean pulmonary vascular abnormalities; these can manifest on imaging as tumefactive perivascular mass-like lesions, pulmonary arteritis, pulmonary arterial aneurysm and stenosis and intravascular filling defect mimicking pulmonary embolism or malignancy [127–129]. Pathologically, the disease is characterised by the presence of dense lymphoplasmacytic infiltration of muscular pulmonary arteries or medium-sized veins, storiform fibrosis and obliterative phlebitis with or without elevated serum IgG4 concentrations. There are case reports showing the advantages of using ^18^F-FDG PET/CT in IgG4-RD to evaluate multiorgan involvement, monitoring treatment response and determine biopsy sites to guide intervention [130–133]. Further studies are needed to define the role of PET/CT and its clinical impact in this group of patients.

### Other PET tracers used in pulmonary vascular imaging

While the ^18^F-FDG tracer is by far the most studied and used PET tracer in PH, several other tracers have been considered and are at various stages of clinical development (figure 1a). 3′-deoxy-3′-[18F]-fluorothymidine (^18^F-FLT), a thymidine analogue, has emerged as a valuable tracer in clinical oncology depicting tumour growth in a variety of malignancies [134] and correlating with histological proliferation markers such as Ki-67 and proliferating cell nuclear antigen [135, 136]. ^18^F-FLT phosphorylation by thymidine kinase 1 leads to ^18^F-FLT retention within the cell, thereby providing a quantitative measurement of proliferating tissue. ^18^F-FLT uptake was increased in the monocrotaline rat model of PH as well as in a small group of eight IPAH patients (figure 2a) [137]. However, when ^18^F-FLT uptake in the human PAH lung was studied in a larger group of patients and compared with an appropriate healthy control group, its use as a biomarker for PAH presence and/or severity could not be validated [138].

^18^F-FDG and ^18^F-FLT were tested in PAH because of their ability to detect proliferative cells in the pulmonary vascular wall. Other tracers were investigated because of their ability to probe other aspects of lung vascular remodelling. Altered transforming growth factor-β type I receptor signalling was detected in pre-clinical models of pulmonary hypertension using the labelled ALK-5 inhibitors [^11^C]-LR111 and [^18^F]-EW-7197 [139]. Increased fibroblast activity was quantified using a gallium-68 [^68^Ga]-labelled fibroblast activation protein, both in the pressure overloaded right heart [140], and in the larger pulmonary arteries of patients with CTEPH [141]. Very recently, macrophage infiltration was imaged in the lungs of monocrotaline rats as well as in the lungs of a pilot group of five PAH patients using the PET tracer ^68^Ga-2-(p-isothiocyanatobenzyl)-1,4,7-triazacyclononane-1,4,7-triacetic acid mannosylated human serum albumin (^68^Ga NOTA-MSA) [142]. Finally, PET imaging has been used in pharmacokinetic studies of PAH-specific drugs, such as labelled phosphodiesterase-5 inhibitors [143] and endothelin receptor antagonists [144], but these tracers have not been explored for clinical application.

## Intravascular ultrasound and optical coherence tomography

Intravascular ultrasound (IVUS) and optical coherence tomography (OCT) are *in vivo* and real-time catheter-based tomographic imaging techniques for identification and delineation of the pulmonary vascular lumen and vessel wall. The resolution of OCT (10–20 μm) is 10-fold higher than that of IVUS (100–150 μm) and hence is better for assessment of segmental and subsegmental vessels. In recent years, both techniques have been used mainly in chronic thromboembolic pulmonary disease as an adjunct to BPA, in which they have been shown to be useful for visualisation and characterisation of lesion morphology, identification of target lesions that may not be easily visible on angiography and evaluation of the immediate effect of BPA [145–147]. In a small study of 31 patients, OCT was found to distinguish distal CTEPH from IPAH [148]. Roik *et al.* [149] demonstrated the improved safety profile and efficacy of BPA in 50 patients using a multimodal combination of IVUS/OCT with pressure wire measurements. However, these results are tempered by the limited sample size and the lack of multicentre validation. Currently, there is no consensus on the use of IVUS and OCT for BPA. Many BPA experts do not advocate their routine usage, particularly as the forceful injection of contrast medium for OCT may increase perfusion pressure in the peripheral pulmonary vasculature and cause pulmonary injury [150].

Numerous publications have shown associations between IVUS- and OCT-derived vessel wall thickness and stiffness and mortality in adult patients with PAH [151–155]. Recently, OCT was used in the paediatric population to demonstrate that wall thickness and diameter are greater in the PH population with significant haemodynamic correlation [156]. PA wall thickness and fibrosis on OCT could also identify early stages of vascular remodelling and identify reverse remodelling after specific vasodilator therapy [157]. IVUS and OCT are promising for prognostication of disease progression and survival in PAH, but larger prospective multicentre trials are needed to assess their clinical impact.

A well-recognised complication of aneurysmal pulmonary artery is the extrinsic compression of left main coronary artery between the dilated pulmonary artery and left coronary sinus. In patients with positive angiographic criteria for extrinsic left main compression, IVUS has been used as a discriminator, as in up to 50% of these patients there may be significant lumen reduction and therefore they can avoid having unneeded coronary stenting [158].

## Micro-CT

Micro-CT is a high-resolution *ex vivo* imaging technique that can help in translation of pre-clinical data into clinical practice. While primarily used in pre-clinical models, this technology is now being applied to human lung samples. Commercially available micro-CT systems use a low-energy micro-focus X-ray source to takes a series of images of an object as it rotates through 360°, resulting in the generation of isotropic 3D images without destroying the sample tissue, generating tomographic data at microscopic spatial resolution (10–200 mm) [159]. Numerous pre-clinical studies have evaluated the vascular changes in PH and the role of chronic hypoxia in the development of PH. Micro-CT of paraffin-embedded human lung samples have outlined pulmonary microvascular anatomy [160], allowing a novel classification of the heterogeneous plexiform lesions [161], and characterised vascular damage and intussusceptive angiogenesis in coronavirus disease 2019 [162].

## Hybrid techniques

### Cardiac FDG-PET and MRI (PET/MR)

Hybrid PET/cardiac magnetic resonance is an emerging technique with great potential to provide morphological, functional and metabolic information from complementary data acquired under the same physiological conditions. Over the past decade, these integrated systems have overcome immense technical challenges including the efficient performance of PET within a magnetic field, maintenance of homogeneity of MRI fields in the presence of a PET detector and the use of MRI attenuation correction maps. Kazimierczyk *et al.* [163] demonstrated the prognostic and predictive information that can be gained in PH using PET-MR; in a small exploratory study involving 26 PAH patients the RV metabolic alterations foreshadowed clinical deterioration and were linked to haemodynamic derangements. As the combination of higher baseline RV glucose uptake and lower RV ejection fraction was associated with worse prognosis, the single hybrid examination provided an opportunity to identify higher risk individuals. The same authors went on to describe how on a follow-up study, augmentation of targeted PAH treatment in these patients with adverse prognostic indicators (baseline specific uptake ratio RV:left ventricle (LV) >1.0 and/or RV ejection fraction <40%) resulted in reversal of unfavourable RV glucose metabolic alteration [164]. Maier *et al.* [165] evaluated 175 patients who were clinically suspected to have cardiac sarcoidosis with PET-MR and identified a subgroup of 33 patients with increased ^18^F-FDG uptake in the wall of the pulmonary artery who were subsequently proven to have PH by right heart catheterisation. This finding offers the potential of subclinical PH detection and provides insights for a potential mechanism of disease progression in sarcoidosis. Not only does PET/MR offer lower radiation exposure compared to PET/CT, it provides optimal co-registration of the two important imaging modalities in cardiac sarcoidosis and PH. Hybrid PET/MR technology has matured over the past few years with promising preliminary evidence, but large-scale prospective validation will be necessary prior to routine clinical use.

### CMR-CPET

A major advantage of CMR is its capability to assess cardiac output during exercise. Using real-time phase-contrast sequence, it is possible to generate a continuous measure of cardiac output with exercise. Proof-of-concept studies in healthy volunteers, PH and congenital heart disease [166, 167] have demonstrated the feasibility of MR-augmented cardiopulmonary exercise testing (CPET) to evaluate abnormal exercise patterns in oxygen uptake, arteriovenous oxygen content gradient for assessment of oxygen extraction and variations in cardiac output. Brown *et al.* [168] used CMR-CPET to study factors contributing to exercise intolerance in patients with systemic sclerosis and showed that the primary contributor to decline in peak oxygen consumption was a reduction in oxygen content gradient and in a subgroup of patients with systemic sclerosis-associated PH, this effect was augmented by a decrease in cardiac output. The tissue oxygen extraction derived by simultaneous measurement of CO and oxygen consumption has the potential to be used as a noninvasive biomarker of functional limitation in systemic sclerosis. One current limitation of CMR-CPET is the use of supine exercise in contradiction to conventional CPET, but nevertheless there seems to be good correlation between the various respiratory metrics derived from CMR and traditional CPET.

### CMR-RHC

Interventional CMR (iCMR) is a composite technique that combines the advantages of CMR and MR-guided RHC. Compared to conventional X-ray fluoroscopy, stand-alone CMR fluoroscopy can provide superior soft tissue visualisation and physiological evaluation without the need for ionising radiation. As real-time CMR imaging can generate frame rates similar to X-ray fluoroscopy, it is also possible to perform integrated flow quantification of systemic and differential pulmonary arterial flows. In the largest published series of paired comparison in 102 patients, Rogers *et al.* [169] demonstrated excellent agreement between iCMR and Fick-derived cardiac output and PVR at baseline, but a better accuracy for iCMR for measurements made during physiological provocation using inhaled 100% oxygen or nitric oxide. In addition to high procedural success rates and excellent safety outcomes of CMR-RHC in adults and children [169, 170], iCMR measurements have also been shown to be more reproducible in RHC in congenital heart disease [171]. While the technical feasibility of CMR-RHC has been established, there are many challenges unique to the MR environment, including the need for interventional devices that would need optimisation for safety and visualisation to mitigate image artifacts and the education and training of multidisciplinary personnel.

A novel approach combining RHC with CMR measurements, to assess individualised therapeutic effects of PAH therapies, is the focus of the PHOENIX study. This study is evaluating remote technology including regulatory approved minimally invasive pulmonary artery pressure monitors (CardioMEMS) and validated CMR end-points should provide valuable insights on the cardiopulmonary impact of therapeutic interventions [172].

## Computational techniques

Computational analysis has emerged as an important component in guiding effective evaluation of PH diagnosis, prognosis and treatment. It allows derivation of quantitative imaging markers, risk-free simulated modelling of response to treatments, and analysis of vast imaging data for AI-/machine learning-based diagnosis and prognosis.

### AI/machine learning

A major development for transforming noninvasive PH diagnosis and management is machine learning and AI tools, thanks to their autonomous analysis of extensive *in vivo* imaging data [173–175]. Recent machine learning analysis of 3D RV motion from CMR showed promise in predicting survival in PH patients, towards enhancing risk differentiation [176]. Machine learning was also shown to hold promise in automating PAH diagnosis from CMR within 10 s image processing time [177]. Radiomics, which are machine learning extracted quantifiable shape and texture image features, from routine CT images can be used to aid in the diagnosis of CTEPH (figure 3a) [178], and can provide complementary phenotypic and prognostic information in systemic sclerosis patients with pulmonary hypertension [179]. Deep learning enabled echocardiography-based RV assessment using automated tracking of the tricuspid annulus in <1 s processing [180]. Deep learning showed promise in predicting RV ejection fraction from apical four-chamber 2D echocardiograms alone, with comparable diagnostic and prognostic performance as 3D echocardiography [181]. Deep learning can automate time-consuming routine measurements, *e.g.* automating quantification of CT scans (figure 3b) [7], and defining contouring for extracting cardiac volumetric data needed for evaluation of ejection fraction, stroke volume, *etc*. [182]. This, in turn, can increase the reproducibility and efficiency to assist clinicians in decision-making. Machine learning and deep learning can also integrate imaging data with clinical reports for more comprehensive evaluation [174], which may aid in the future in PH screening and diagnosis.

A large study using machine learning to classify CMR features [8] demonstrated improved prediction of mortality compared to the Registry to Evaluate Early and Long-term PAH Disease Management (REVEAL) method, and also that end-systolic interventricular septum and end-diastolic LV abnormalities indicated the highest risk of mortality. Given the widespread availability of CTPA and its increasing use in the assessment of unexplained breathlessness there is interest in the use of CT metrics as a noninvasive diagnostic tool to identify patients with PH. Swift *et al.* [183] have demonstrated that CT features including a pulmonary artery ≥30 mm, RV outflow tract diameter ≥6 mm and RV:LV ratio ≥1 are highly predictive of the presence of PH. Utilising AI it is now possible to automatically segment large vessels and cardiac chambers allowing for a fully automated assessment of pulmonary artery pressure based on CT features [6]. A different approach would be to risk stratify patients with PAH based on clinical and imaging parameters, as proposed by Sonnweber *et al.* [184]. Their study evaluated machine learning tools based on several parameters (age, 6MWD, red blood cell distribution width, cardiac index, PVR, NT-proBNP and right atrial area). This tool was highly able to predict mortality risk according to clinical phenotyping [184].

Several publications have evaluated the role of AI for the detection of pulmonary emboli [185–187]. A prospective study tested a commercially available system and could not demonstrate significant diagnostic improvement (although this study was relatively small), but did show improved reporting speed [185]. A large retrospective study using batch processing of commercially available AI algorithm did demonstrate improved accuracy with improved sensitivity from 91.6% to 96.8%, and specificity from 99.7% to 99.9% [186]. Lastly, a recent implementation of AI software was able to increase the detection of incidental pulmonary embolism in cancer patients from 0.8% to 2.6% with improved reporting and treatment times [187].

There are many more attempts to bring in AI tools to enhance imaging-based and clinical parameter-based assessment in pulmonary vascular disease, but most are still in their infancy and will require prospective validation studies. The effectiveness of machine learning and AI models highly depends on the quality and diversity of the training data. Generalisability of models to unseen data remains a bottleneck and many AI decisions can be considered a black box with potential for unpredictable “hallucinations” [175, 188]. Bias in AI models, *e.g.* to gender and race, remains concerning [189]. Increasing AI explainability is critical to increase clinicians' trustworthiness in such models [188]. As it stands, machine learning and AI should be considered primarily as assistive tools in the arsenal of the clinicians’ toolkit rather than a standalone magic solution. Ultimately, the clinician should make the final decision based on a comprehensive patient data assessment that may involve, but should not be limited to, AI tools.

### Computational fluid dynamics

One powerful application of computational techniques is computational fluid dynamics (CFD) that leverages physics, mathematics and patient-specific data to simulate complex PH pathophysiology and predict treatment outcomes. CFD models provide noninvasive measurements of pressure gradients, flow dynamics, and WSS [190–193]. CFD analysis, using patient-specific 3D MRI data, found significantly lower central pulmonary artery WSS in PAH patients compared to controls [194]. Likewise, 3D CFD simulations with patient-specific CT-derived geometry in mixed PH patients (WSPH groups 2, 3 and 4) showed correlations between WSS with PVR, and with arterial compliance [195]. A comprehensive patient-specific 3D CFD model in PAH patients, including vascular and ventricular dynamics along with ventricular strain, showed a strong inverse correlation between peak RV free wall contractility and the RV/LV end-diastolic volume ratio [196]. In CTEPH patients, 3D CFD simulations of treatment response using patient-specific geometry from CT angiography data suggested potential benefits for both pulmonary endarterectomy and BPA in improving PA blood flow dynamics including WSS and reducing blood flow stagnation [197].

Despite the potential of CFD modelling, it remains computationally demanding and its accuracy relies on the quality of incorporated patient data and underlying assumptions, such as boundary conditions, which simplify the true complex flow–structure and vascular–ventricular interactions. Hence, CFD results should be cautiously interpreted within the broader clinical context, in conjunction with other *in vivo* patient data, and not solely relied upon to make decisions in isolation. Recent developments in *in vivo* imaging techniques, such as 4D flow MRI, have enabled the direct measurement of noninvasive blood flow velocities and the derivation of advanced haemodynamic parameters, including WSS [69], kinetic energy [72], viscous energy loss [198], pressure gradients [74], vortex flow [70, 71] and vorticity [72]. Consequently, there is an opportunity for improved integration of mathematical modelling with *in vivo* data, potentially overcoming or mitigating the limitations associated with CFD modelling. For instance, a recently developed 4D virtual catheter technique in the thoracic aorta, which integrates mathematical modelling with 4D flow MRI to directly measure advanced haemodynamics *in vivo* might hold potential for evaluating PH [72].

## Integrated approach with the RV

Although the primary focus of this review is on the pulmonary vasculature in PH, it is the response of the RV to this disease that ultimately dictates patient outcomes [199]. As RV afterload increases in PH, the RV attempts to compensate to maintain coupling (maximum stroke work efficiency) with the pulmonary system; a relationship that underlies the concept of the RV–PA unit. Therefore, a comprehensive understanding of the mechanisms that drive PH must include a detailed assessment of this entire unit. Fortunately, many of the imaging modalities described earlier can also provide mechanistic insight into the various components of the heart in addition to the lungs. Table 2 highlights imaging modalities and the data each one is able to provide for both the pulmonary vasculature and the RV.

**TABLE 2:** Integrating cardiac and pulmonary imaging modalities in the evaluation of pulmonary hypertension

	Pulmonary vasculature	Right ventricle
**Echo**	Mean PA pressure PA systolic pressure PA diastolic pressure PA wedge pressure Pulmonary vascular resistance RV–PA coupling	RV end-diastolic pressure Chamber size and function Interventricular septal flattening Congenital defects Strain analysis RV–PA coupling
**CT**	Parenchymal disease Pulmonary vascular pruning Intraparenchymal vascular volumes Anatomic thrombus distribution Pulmonary vascular perfusion Pulmonary vascular microvasculopathy Systemic collateral circulation assessment Coronary artery evaluation	Chamber size and function RV mass Interventricular septal flattening Congenital defects
**PET**	Pulmonary vascular proliferation Pulmonary vascular inflammation Pulmonary vascular fibroblast activation Pulmonary vascular macrophage infiltration Sympathetic innervation	Chamber size and function Perfusion Metabolism Inflammation
**MRI**	Lung perfusion/ventilation Angiography (with/without contrast) PA mean transit time PA pulsatility index PA vortex flow PA vorticity PA helical flow PA wall shear stress Kinetic energy Viscous energy loss Pressure gradients	Chamber size and function Ventricular mass Interventricular septal flattening Congenital defects Replacement fibrosis Diffuse fibrosis Strain analysis Flow dynamics Spectroscopy Perfusion

CT: computed tomography; PET: positron emission tomography; MRI: magnetic resonance imaging; PA: pulmonary artery; RV: right ventricle.

Echocardiography is the first-line imaging modality in evaluating the RV and identifying possible PH. It excels at estimating haemodynamics and can help differentiate pulmonary arterial from pulmonary venous hypertension. However, its evaluation of the RV is constrained due to the geometric complexity of the chamber, and it falls short in identifying lung pathologies due to resolution limitations [199]. Lung ultrasound is emerging as a potential point-of-care diagnostic tool for both pleural and parenchymal disease, but interpretation of these images can be challenging, with operator inexperience being a notable limitation.

CT is particularly adept at providing an integral view of the cardiopulmonary unit [66]. CT is often the first imaging modality that hints at the presence and severity of PH, through its visualisation of an enlarged right side of the heart, increased pulmonary artery diameter and presence or absence of pericardial fluid and contrast backflow into the inferior vena cava. At the same time, CT can be used to identify the causes of PH, such as parenchymal lung disease, congenital heart disease, an enlarged left atrium and acute or chronic thrombi. Newer technology provides noncontrast options for detecting pulmonary pathology and dual-energy CT allows the visualisation of the functional consequences of CTEPH. While retrospectively gated CT provides accurate measurements of RV size and function, radiation exposure limits its utility for this purpose.

In PH, PET offers functional information by assessing perfusion, inflammation and metabolism in both the lungs and the heart. This makes PET unique compared to the other imaging modalities in that it has the potential to identify earlier stages of PH before adverse remodelling has occurred. From a clinical perspective, PET, much like CT, involves radiation exposure and its cost limits availability for many patients.

CMR provides multiparametric data and remains the gold standard for measuring cardiac chamber size and function. Both 2D and 4D flow MRI allow for haemodynamic measurements virtually anywhere in the heart without the use of contrast. Tissue characterisation, pulmonary angiography and perfusion of both the lungs and the heart (figure 2c) offer new insights into the pathology of PH and potential targets for future therapies.

## Conclusion

Pulmonary hypertension remains a challenging condition to diagnose, classify and treat. Despite advances in therapies and improved outcomes over the past two decades, the time to diagnosis remains unchanged. The emergence of previously underreported phenotypes has underlined the need to improve assessment and classification. While the development of therapies that target biological pathways implicated in the pathogenesis of PAH has highlighted the deficiency of currently use clinical trial end-points that are poor surrogates for pulmonary vascular remodelling.

Significant progress has been made using imaging modalities such as CT and MRI in the evaluation of patients with PH; however, the international community has been slow to incorporate advances in imaging into guidelines and recommendations. In significant part this has been due to the nature of studies, often retrospective, in single centres, with small numbers, frequently without external validation, using semi-quantitative techniques which are frequently not standardised and perceived concerns regarding the availability of newer imaging technology and their translation into routine clinical practice. However, over the past decade the international community has recognised some of these deficiencies resulting in more collaboration and the conduct of larger multicentre imaging studies.

In this review we have highlighted further advances in imaging technology, including the application of AI techniques. Despite this a number of challenges remain including a need to 1) standardise approaches, particularly for emerging imaging techniques; 2) establish and refine normative ranges, recognising the impact of age, sex and gender; 3) establish minimally important clinical differences for CT and MRI metrics to evaluate interventions, benchmarked to meaningful clinical end-points; 4) perform further large multicentre studies to compare current and emerging imaging techniques to improve diagnosis and disease assessment; 5) conduct cost-effectiveness analysis of alternative imaging approaches; 6) refine techniques to improve assessment of the distal pulmonary vasculature and gas exchange; and 7) further develop AI approaches to ensure actionability of imaging findings in clinical practice.

Imaging has the potential to further improve outcomes by reducing time to diagnosis, refining classification, aiding the assessment of new interventions and improving our understanding of disease mechanisms. By improving collaboration between physicians, radiologists, physicists, scientists, patients and industry we are optimistic this will be achieved.

## Shareable PDF

10.1183/13993003.01128-2024.Shareable1 This one-page PDF can be shared freely online.
Shareable PDF ERJ-01128-2024.Shareable
