# Supplementary material for: Emerging multimodality imaging techniques for the pulmonary circulation
Source: Eur Respir J. 2024 Oct 31;64(4):2401128. doi: 10.1183/13993003.01128-2024 (PMC11525339; doi:10.1183/13993003.01128-2024)
Supplement: Supplementary file 1 [file ERJ-01128-2024.Shareable.pdf]

EUROPEAN RESPIRATORY JOURNAL
TASK FORCE REPORT
S. RAJAGOPAL ET AL.

SHAREABLE PDF

Emerging multimodality imaging techniques for the pulmonary circulation

Sudarshan Rajagopal[1], Harm J. Bogaard[2], Mohammed S.M. Elbaz[3], Benjamin H. Freed[4], Martine Remy-Jardin[5], Edwin J.R. van Beek[6], Deepa Gopalan[7] and David G. Kiely[8]

[1]Department of Medicine, Duke University School of Medicine, Durham, NC, USA. [2]Department of Pulmonology, Amsterdam University Medical Center, Location VU Medical Center, Amsterdam, The Netherlands. [3]Department of Radiology, Northwestern University Feinberg School of Medicine, Chicago, IL, USA. [4]Feinberg School of Medicine, Northwestern University, Chicago, IL, USA. [5]IMALLIANCE-Haut-de-France, Valenciennes, France. [6]Edinburgh Imaging, Queens Medical Research Institute, University of Edinburgh, Edinburgh, UK. [7]Department of Radiology, Imperial College Healthcare NHS Trust, London, UK. [8]Sheffield Pulmonary Vascular Disease Unit and NIHR Biomedical Research Centre Sheffield, Royal Hallamshire Hospital, Sheffield, UK.

Corresponding author: Deepa Gopalan (d.gopalan@nhs.net)

Check for updates

Shareable abstract (@ERSpublications)
Innovative imaging can enhance our understanding of pulmonary vascular disease mechanisms, refine classification, improve outcomes by reducing time to diagnosis, aid treatment decision-making and provide a holistic approach to assess impact to therapies. https://bit.ly/3RvMWTQ

Cite this article as: Rajagopal S, Bogaard HJ, Elbaz MSM, *et al*. Emerging multimodality imaging techniques for the pulmonary circulation. *Eur Respir J* 2024; 64: 2401128 [DOI: 10.1183/13993003.01128-2024].

This extracted version can be shared freely online.

Copyright ©The authors 2024.

This version is distributed under the terms of the Creative Commons Attribution Licence 4.0.

This article has an editorial commentary: https://doi.org/10.1183/13993003.01222-2024

Received: 11 June 2024
Accepted: 11 June 2024

Abstract

Pulmonary hypertension (PH) remains a challenging condition to diagnose, classify and treat. Current approaches to the assessment of PH include echocardiography, ventilation/perfusion scintigraphy, cross-sectional imaging using computed tomography and magnetic resonance imaging, and right heart catheterisation. However, these approaches only provide an indirect readout of the primary pathology of the disease: abnormal vascular remodelling in the pulmonary circulation. With the advent of newer imaging techniques, there is a shift toward increased utilisation of noninvasive high-resolution modalities that offer a more comprehensive cardiopulmonary assessment and improved visualisation of the different components of the pulmonary circulation. In this review, we explore advances in imaging of the pulmonary vasculature and their potential clinical translation. These include advances in diagnosis and assessing treatment response, as well as strategies that allow reduced radiation exposure and implementation of artificial intelligence technology. These emerging modalities hold the promise of developing a deeper understanding of pulmonary vascular disease and the impact of comorbidities. They also have the potential to improve patient outcomes by reducing time to diagnosis, refining classification, monitoring treatment response and improving our understanding of disease mechanisms.
